# Genome-wide identification and functional characterization of *PP2C* genes in the wild relative of sweet potato *Ipomoea trifida*

**DOI:** 10.1186/s12870-025-07764-4

**Published:** 2025-12-29

**Authors:** Yawei Li, Shouchen Qiao, Yumeng Yin, Yannan Wang, Dandan Xu, Qianqian Bian, Zhihe Kang, Guozheng Cao, Guorui Zhao, Junxia Li, Yufeng Yang

**Affiliations:** 1 Cereal Crop Research Institute, Henan Academy of Agricultural Sciences, Zhengzhou, 450002 China; 2 Tongxu County Institute of Agricultural Science, Tongxu, 475400 China

**Keywords:** *PP2C* gene family, *Ipomoea trifida*, Drought stress, Stem nematode, ABA signaling pathway

## Abstract

**Background:**

Protein phosphatase 2 C (PP2C) proteins play crucial roles in plant growth, development, and stress responses. However, *PP2C* gene family members in *Ipomoea trifida* (wild relative of sweet potato) have not been comprehensively investigated, thereby limiting our understanding of their functions.

**Results:**

We identified 91 *PP2C* genes (*ItfPP2C1–91*) that were unevenly distributed across all 15 *I. trifida* chromosomes. On the basis of a phylogenetic analysis, these genes were classified into 13 subfamilies, with subfamilies E, A, and D containing the most genes. Conserved motif and gene structure analyses revealed subfamily-specific patterns, with motif 2 identified as the most conserved motif. Numerous promoter cis-acting elements related to hormone responses and stress tolerance were identified. Tissue-specific *ItfPP2C* expression patterns were observed, with several genes expressed at high levels in all examined tissues, while other genes were expressed in specific tissues. Under drought conditions, most *PP2C* genes had upregulated expression levels, with significant increases in *ItfPP2C15*, *16*, *30*, and *77* (subfamily A) expression suggesting that they may be key drought-responsive candidate genes. In response to a stem nematode infection, *ItfPP2C30*, *ItfPP2C77*, and *ItfPP2C89* were differentially expressed between resistant and susceptible varieties. Moreover, *ItfPP2C90* expression was significantly induced in Z22 and L9 at 12 h. Hence, these genes may be involved in disease resistance. A protein interaction network analysis identified proteins that may interact with ItfPP2C30 and ItfPP2C77, most of which were ABA receptors (PYLs) and kinases (SnRK2s).

**Conclusions:**

Our comprehensive analysis of the *PP2C* gene family in *I. trifida* provides valuable insights into their evolutionary relationships, structural features, and potential functions related to growth and stress responses. *ItfPP2C30* and *ItfPP2C77* were identified as promising candidates for improving both drought and nematode resistance, with the encoded proteins potentially interacting with PYL and SnRK2 proteins in stress-related signaling pathways.

**Supplementary Information:**

The online version contains supplementary material available at 10.1186/s12870-025-07764-4.

## Background

Protein phosphatases (PPs) are critical regulators of reversible protein phosphorylation, a post-translational modification that governs many cellular signaling events in plants [1, 2]. Among the major classes of PPs, the protein phosphatase 2 C (PP2C) family is evolutionarily conserved and plays indispensable roles in plant growth, development, and stress adaptation [3, 4]. PP2Cs are Mg^2+^/Mn^2+^-dependent serine/threonine phosphatases that function primarily as negative regulators of protein kinase signaling pathways [5, 6]. PP2Cs have been extensively studied in model species, such as *Arabidopsis thaliana* and *Oryza sativa*, in which they modulate hormone signaling (e.g., abscisic acid [ABA], auxin, and jasmonic acid), biotic and abiotic stress responses, and developmental processes, including seed germination and stomatal closure [7–12].

PP2C proteins are characterized by a conserved catalytic domain consisting of 11 motifs that form a characteristic α/β fold [13]. In *A. thaliana*, the PP2C family comprises 80 members that are phylogenetically divided into 13 subfamilies (A–L, with subfamily F subdivided into F1 and F2) according to sequence homology and functional divergence [14]. Members of PP2C subfamily A, such as ABI1, ABI2, and HAB1, are well characterized as central negative regulators of ABA signaling that interact with ABA receptors (PYR/PYL/RCAR) and SnRK2 kinases to desensitize stress responses [15–17]. Other subfamilies, including B, C, and D, are involved in regulating mitogen-activated protein kinase (MAPK) pathways, cell cycle progression, and responses to biotic stresses [4, 18, 19].

Abiotic stresses, such as drought, salinity, and extreme temperatures, pose significant challenges to global crop productivity. PP2Cs serve as key regulators of signaling networks, particularly through ABA-mediated pathways [4, 20, 21]. Under drought conditions, ABA accumulates and binds to PYR/PYL receptors, leading to the inhibition of subfamily A PP2Cs. PP2Cs release SnRK2 kinases to phosphorylate downstream targets, including transcription factors (e.g., ABF/AREB) and anion channels (e.g., SLAC1), thereby promoting stomatal closure and stress-responsive gene expression [22–25]. Loss-of-function mutations in *A. thaliana* subfamily A PP2Cs (e.g., ABI1 and HAB1) result in enhanced drought tolerance, highlighting their potential utility as targets for crop improvement [26, 27]. In addition to their contributions to abiotic stress responses, PP2Cs can also modulate plant immunity against pathogens and pests. Subfamily B PP2Cs, such as AP2C1 and PP2C5, negatively regulate MAPK pathways activated by pathogen-associated molecular patterns, leading to suppressed defense responses [18, 28]. PP2C38 negatively regulates immune responses in *A. thaliana* by controlling the phosphorylation and activation status of BIK1 [29]. However, PP2C functions in biotic stress responses are not as thoroughly characterized as their functions during abiotic stress responses, particularly in non-model crops.

Sweet potato (*Ipomoea batatas* (L.) Lam), which is an important food and industrial crop cultivated worldwide, is valued for its high yield, rich nutritional content, and ability to grow in marginal environments [30, 31]. Its productivity is threatened by abiotic stresses (e.g., drought) and biotic stresses (e.g., stem nematode infections), which can decrease yields by up to 50% [32]. The genetic improvement of sweet potato is hindered by its complex hexaploid genome (2n = 6x = 90) and limited genomic resources [33, 34]. *Ipomoea trifida* (2n = 2x = 30), a diploid wild relative of sweet potato, serves as a key model for genetic studies because of its simpler genome and close phylogenetic relationship to cultivated sweet potato [35, 36]. The release of the *I. trifida* reference genome has enabled genome-wide analyses of gene families, including those comprising stress-responsive genes [34]. To date, PP2C family members have been identified in various crops, such as rice [14], maize [37], and tomato [38, 39], but they have not been comprehensively characterized in *Ipomoea* species.

In this study, 91 PP2Cs were identified in *I. trifida* and classified into 13 subgroups. We systematically investigated the identified PP2Cs in terms of their protein characteristics as well as the chromosomal localization, phylogenetic relationships, conserved motifs, structures, and promoter cis-acting elements of the corresponding genes in *I. trifida*. Moreover, the tissue specificity and expression patterns of genes encoding PP2Cs responsive to drought and stem nematode infections were analyzed by RNA-seq. Four candidate genes related to responses to drought and stem nematode infections were identified, among which *ItfPP2C30* and *ItfPP2C77* were associated with both stresses. On the basis of a protein interaction network analysis, ItfPP2C30 and ItfPP2C77 may synergistically regulate drought and stem nematode-induced stress responses by binding to core components of ABA signaling pathways (i.e., PYLs and SnRK2s). These findings provide relevant insights into the evolutionary and functional diversification of PP2Cs in *Ipomoea*, with potential implications for developing stress-tolerant sweet potato varieties via molecular breeding.

## Methods

### Data resources

Whole-genome information and genome annotation files for *I. trifida* were obtained from the Sweetpotato Genomics Resource (http://sweetpotato.plantbiology.msu.edu/, accessed on 1 January 2025). The PP2C family Hidden Markov Model (HMM) profile (PF00481) was downloaded from the InterPro database (https://www.ebi.ac.uk/interpro/, accessed on 4 March 2025). *A. thaliana* PP2C proteins were downloaded from the TAIR database (https://www.arabidopsis.org/, accessed on 5 March 2025). The longest transcript and corresponding protein sequence were determined using TBtools (v2.225) software.

### Genome-wide identification of *PP2C* gene family members in *I. trifida*

To accurately identify *PP2C* gene family members in *I. trifida*, the sequences of 80 AtPP2C proteins were used as queries for a BLASTP search (E-value < 1 × 10^− 5^) using TBtools software. Subsequently, the *PP2C* family HMM profile was used to search for candidate proteins with conserved *PP2C* domains using the Simple HMMER Search program in TBtools software. Finally, all putative *PP2C* proteins were confirmed using the NCBI CDD-search tool (https://www.ncbi.nlm.nih.gov/Structure/bwrpsb/bwrpsb.cgi, accessed on 7 March 2025) and the Simple Modular Architecture Research Tool (SMART) (http://smart.embl-heidelberg.de/, accessed on 7 March 2025). Sequences that did not contain the complete *PP2C *domain were eliminated to reveal the final *PP2C *proteins in *I. trifida*.

### Chromosomal distribution and bioinformatics analysis of the *ItfPP2C* gene family

Physical positions and chromosomal distributions of 91 *ItfPP2C* genes were provided by the corresponding genome annotation files (GFF3). TBtools software was used to visualize the chromosomal distribution of *ItfPP2C* genes. The following characteristics of the proteins encoded by the identified *ItfPP2C* genes were determined using TBtools software: size, molecular weight, theoretical isoelectric point (pI), instability index, aliphatic index, and grand average of hydropathicity (GRAVY) value. The online tool Cell-PLoc (http://www.csbio.sjtu.edu.cn/bioinf/Cell-PLoc-2/) was used to predict the subcellular localization of *ItfPP2C *proteins.

### Subcellular localization

The full-length CDS of the ItfPP2C77 were cloned to generate the 35 S::ItfPP2C77-GFP construct. The construct was subsequently introduced into 4-week-old tobacco leaves via *A. tumefaciens* strain GV3101-mediated transformation. The subcellular localization of the fusion protein was examined using confocal laser-scanning microscopy.

### Multiple sequence alignment and phylogenetic analysis of *ItfPP2Cs*

Full-length *PP2C *protein sequences from *I. trifida* and *A. thaliana* were aligned using ClustalW. A phylogenetic tree was constructed according to the neighbor-joining method using MEGA6, with 1,000 bootstrap replicates. The final phylogenetic tree was visualized using the online software iTOL (http://itol.embl.de/).

### Analyses of *ItfPP2C* gene structures and conserved motifs

Motifs predicted by the MEME online tool along with the results of the phylogenetic analysis, GFF3 files, and *ItfPP2C* genes were submitted to TBtools to analyze gene structures and visualize conserved motifs. The number of motifs was set at eight, with the size of each motif set at 6–50 bp.

### Analysis of *ItfPP2C* promoter cis-acting elements

The 2-kb upstream region of *ItfPP2C* genes was submitted to PlantCARE (http://bioinformatics.psb.ugent.be/webtools/plantcare/html/) to predict promoter cis-acting elements, which were visualized using TBtools software.

### Analysis of *ItfPP2C* expression levels in various tissues

*ItfPP2C* expression data were downloaded from the Sweet Potato Genomics Resource (http://sweetpotato.plantbiology.msu.edu/, accessed on 10 January 2025). *ItfPP2C* expression levels were determined in terms of fragments per kilobase of exon per million fragments mapped (FPKM) values. A heat map of *ItfPP2C* expression levels was constructed using TBtools software.

### Analysis of *ItfPP2C* expression in response to drought and stem nematode infection

*ItfPP2C* expression data in response to drought and stem nematode infection were from our previously published studies [40, 41]. For both datasets, each experimental group (including different stress treatments, and time points) was subjected to three biological replicates to ensure statistical reliability. The sequencing output yielded approximately 6Gb of clean data per sample. The quality of the sequencing data was rigorously assessed, with Q20 and Q30 scores both exceeding 90% for all libraries. Subsequent bioinformatic analyses were conducted based on these high-quality data.

In the drought treatment, drought-sensitive variety, i.e., Jinong432, and drought-tolerant variety, i.e., Zhenghong23 were used. Four treatments were set, namely, CK (control), D1 (mild drought), D2 (moderate drought), and D3 (severe drought). In the stem nematode infection treatment, stem nematode-sensitive variety, i.e., Longshu9, and stem nematode-tolerant variety, i.e., Zhenghong22 were used. Six treatments, namely 0 h, 12 h, 1 d, 3 d, 10 d, and 30 d after infection with stem nematodes were set.

### qRT-PCR analysis

Total RNA was extracted from fibrous roots, storage roots, stems, petioles, and leaves of Zhenghong22 using the RNA-prep Pure Plant Kit (DP419, Tiangen, Beijing, China) following the manufacturer’s instructions. First-strand cDNA was synthesized using the PrimeScript™ RT reagent Kit (RR047A, TaKaRa, Japan) according to a standard protocol. qRT-PCR was performed using Tag Pro Universal SYBR qPCR Master Mix (Q712, Vazyme, Nanjing, China). The PCR amplification was initiated with a denaturation step at 95 °C for 3 min, followed by 40 cycles comprising 95 °C for 10 s and 60 °C for 30 s. Three biological replicates were performed for each analysis. The relative expression level for each gene was calculated using 2^−ΔΔCt^ method. All primers were designed using the NCBI Primer-BLAST tools (Table S1).

### Prediction of proteins that interact with ItfPP2C30 and ItfPP2C77

SMART (http://smart.embl-heidelberg.de/) was used to identify conserved domains in ItfPP2C30 and ItfPP2C77. ItfPP2C30 and ItfPP2C77 protein sequences were submitted to STRING12.0 (https://cn.string-db.org/) to predict their interacting proteins. Integrated data for known and predicted interactions for the model organism *A. thaliana* were used as the reference. The minimum interaction score was set at the highest confidence level (> 0.9). Functional annotations of the predicted interacting proteins were downloaded from the TAIR website (https://www.arabidopsis.org/).

## Results

### Identification and characterization of *ItfPP2C* genes

**Fig. 1:**
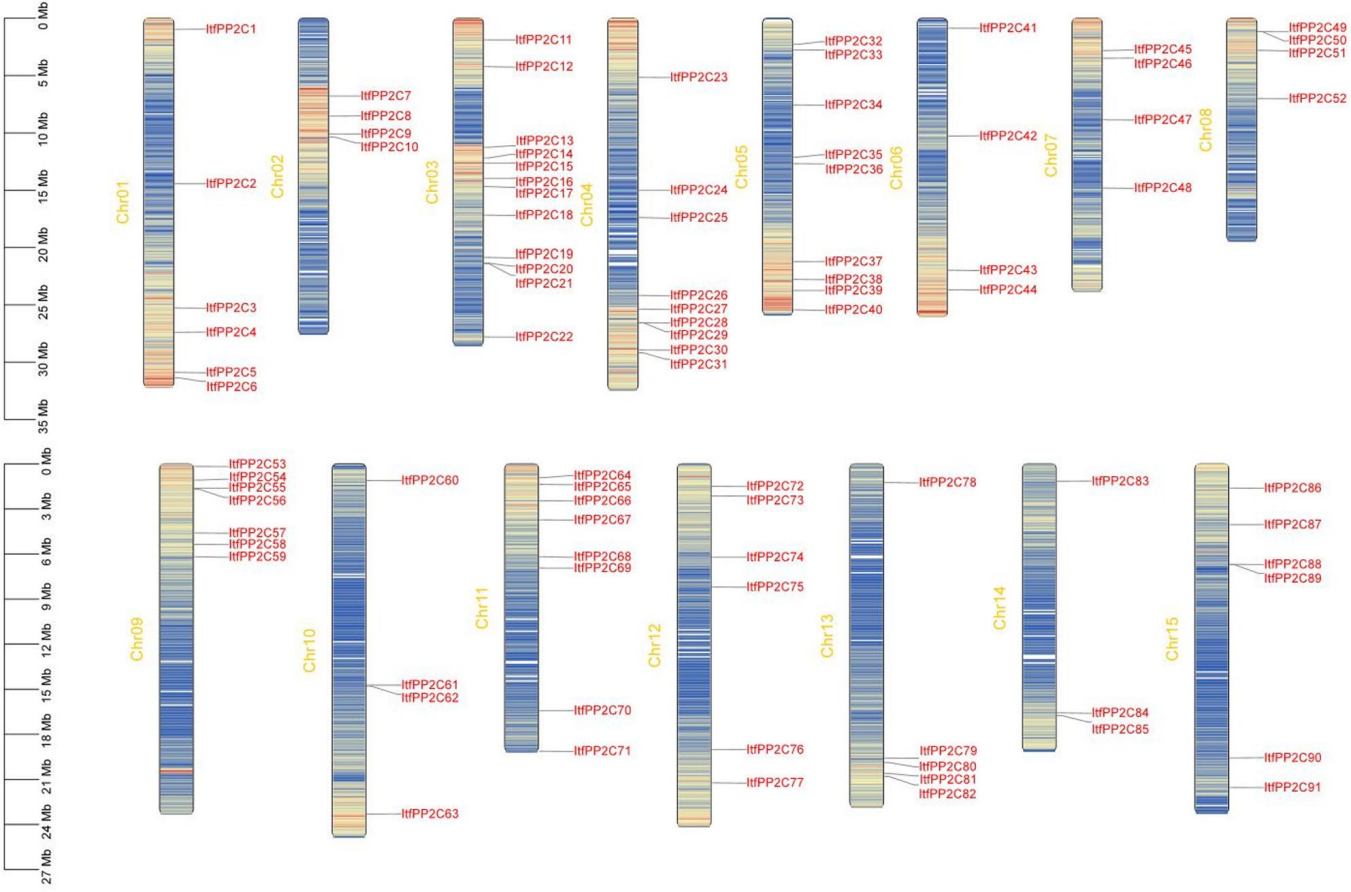
Chromosomal localization and distribution of *PP2Cs* in *I. trifida*. The bars represent chromosomes. The chromosome numbers are displayed on the left side, and the gene names are displayed on the right side

A total of 91 *PP2C* genes were identified in the whole genome of *I. trifida* and then named *ItfPP2C1*–*ItfPP2C91* on the basis of their chromosomal locations. The 91 *ItfPP2C* genes were unevenly distributed across all 15 chromosomes in *I. trifida*, with 12 genes on chromosome 03, nine genes on chromosomes 04 and 05, eight genes on chromosome 11, seven genes on chromosome 09, six genes on chromosomes 01, 12, and 15, five genes on chromosome 13, four genes on chromosomes 02, 06, 07, 08, and 10, and three genes on chromosome 14. Notably, although chromosomes 02, 06, 07, 08, and 10 contained four *ItfPP2C* genes each, the distribution of these genes on the chromosomes differed (Fig. 1).

The basic characteristics of *PP2C *proteins were analyzed according to their sequences. The putative proteins consisted of 248–1,080 amino acids, with a molecular weight of 27,688.5–121,852.8 Da and a theoretical pI of 4.18–9.12 (Table S2). Their instability and aliphatic indices were 28.65–70.63 and 64.42–94.45, respectively (Table S2). The GRAVY value of most *ItfPP2C* proteins was less than 0, indicating that they are hydrophilic proteins; the exception was ItfPP2C51, which had a GRAVY value of 0.04 (Table S2). According to a subcellular localization analysis of the 91 *ItfPP2C *proteins, 59 were predicted to be localized in the nucleus, 13 were localized in the chloroplast, 14 were localized in both the chloroplast and nucleus, and five (5.5%) were localized in other subcellular locations, including the cell membrane, cell wall, cytoplasm, and peroxisome (Table S2).

**Fig. 2:**
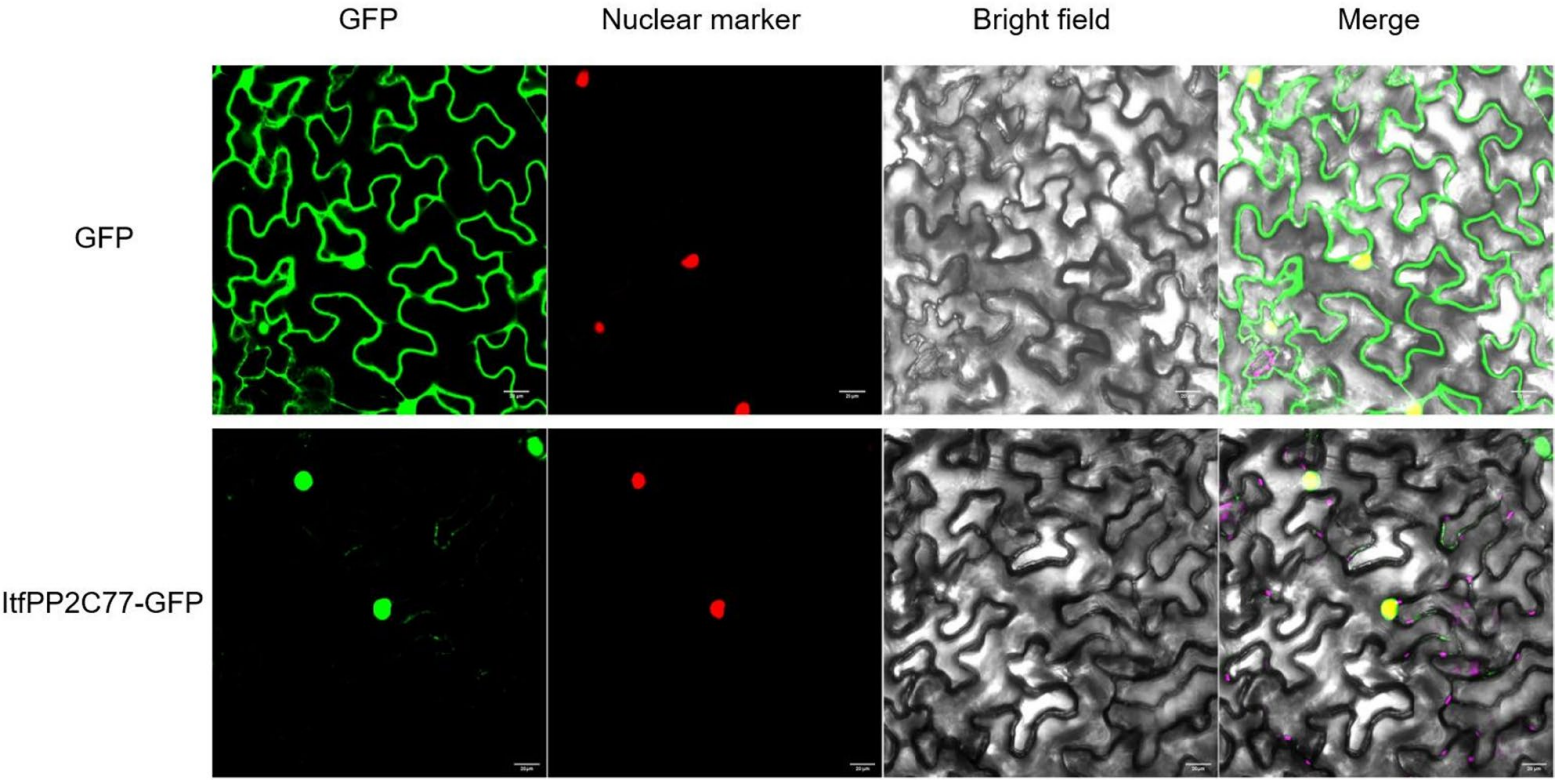
Subcellular localization of ItfPP2C77 in tobacco leaves. GFP was fused to the C-terminus of ItfPP2C77. Fluorescence was observed at 48 h post-infiltration. Scale bars, 20 μm

To experimentally validate the subcellular localization predicted by bioinformatic analysis, we selected *ItfPP2C77* for confirmation. The full-length coding sequence of *ItfPP2C77* was fused to the N-terminus of GFP under the control of the CaMV 35 S promoter. As shown in Fig. 2, the green fluorescence signal of the ItfPP2C77-GFP fusion protein was exclusively localized to the nucleus, and completely overlapped with the nuclear marker signal, confirming its nuclear localization. In contrast, the signal from the GFP control was distributed throughout the entire cell, including the nucleus and cytoplasm. These results provide experimental evidence that ItfPP2C77 is a nuclear-localized protein, consistent with our bioinformatic prediction.

### Phylogenetic relationships of *PP2C* genes in *I. trifida* and *A. thaliana*

**Fig. 3:**
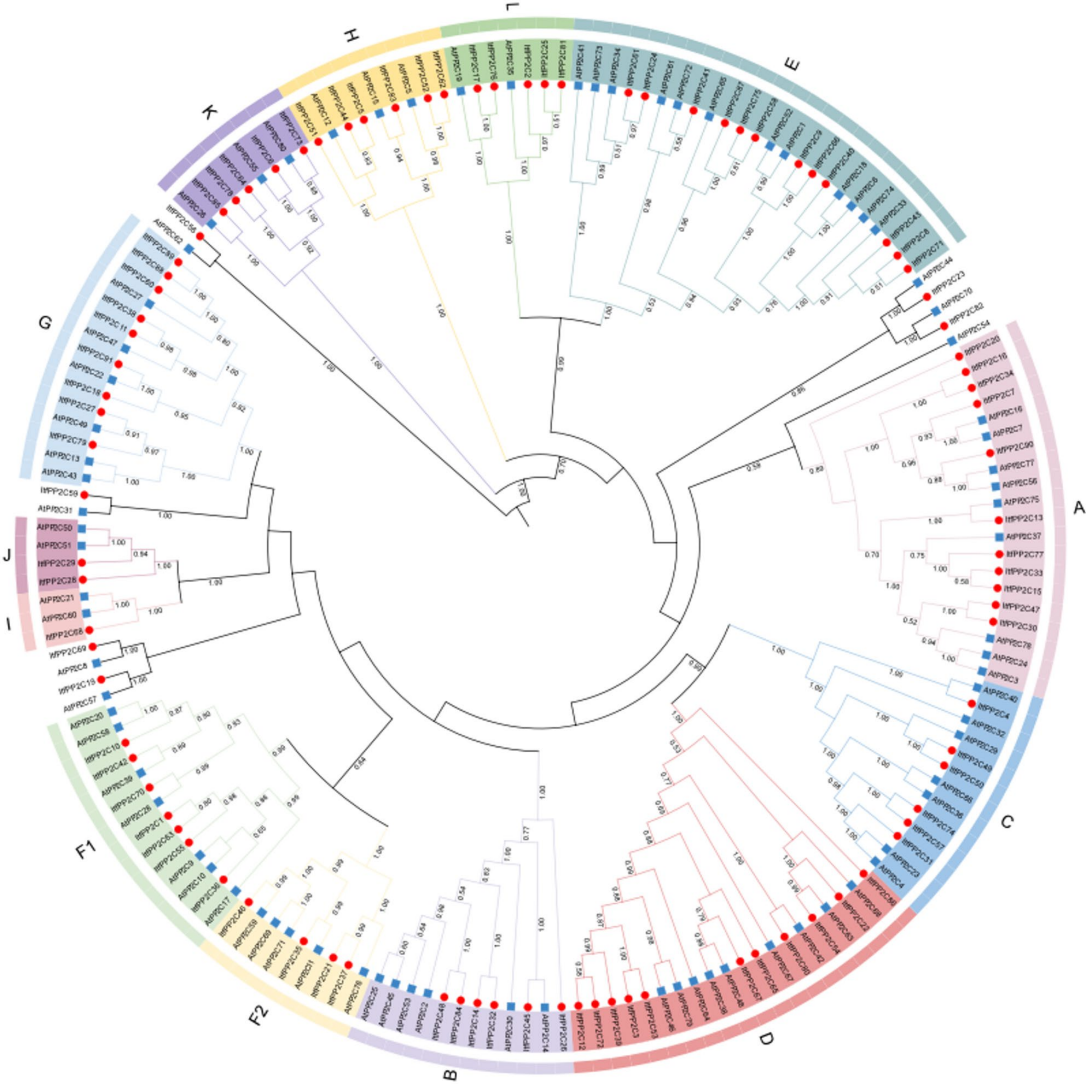
Phylogenetic tree of *PP2C* family members between *I. trifida* (Itf) and *A. thaliana* (At). The phylogenetic tree was constructed using MEGA 6 based on “Neighbor-Joining” and bootstrap analysis, numbers at nodes are bootstrap support values (1000 replicates). Except for the ungrouped *PP2C* proteins, all *PP2Cs *members were classified into 13 subfamilies (**A**–**L**), each represented by a different color. The members of the F subfamily are divided into two subfamilies, named F1 and F2, respectively

To clarify the functional and evolutionary relationships of *PP2C* gene family members, a phylogenetic tree was constructed on the basis of a multiple sequence alignment of 91 *ItfPP2C *proteins in *I. trifida* and 80 AtPP2C proteins in *A. thaliana* (Fig. 3). The *ItfPP2C *proteins were unevenly grouped into 13 subfamilies (A–L, with subfamily F divided into F1 and F2), but ItfPP2C19, 23, 56, 59, 69, and 82 were not grouped in any subfamily. Each subfamily contained at least one *PP2C *from *I. trifida* and *A. thaliana.* Subfamily E had the most *ItfPP2C *proteins (12), whereas subfamilies A and D had 11 *ItfPP2C *proteins, subfamilies B, C, and H had six *ItfPP2C *proteins, and subfamilies K and L had five *ItfPP2C *proteins. Subfamilies F1, F2, G, I, and J had seven, four, nine, one, and two *ItfPP2C *proteins, respectively. This classification was consistent with that of *PP2Cs *in *A. thaliana* and rice, suggesting that *ItfPP2C* genes were highly conserved during evolution.

### *ItfPP2C* gene structures and conserved motifs

**Fig. 4:**
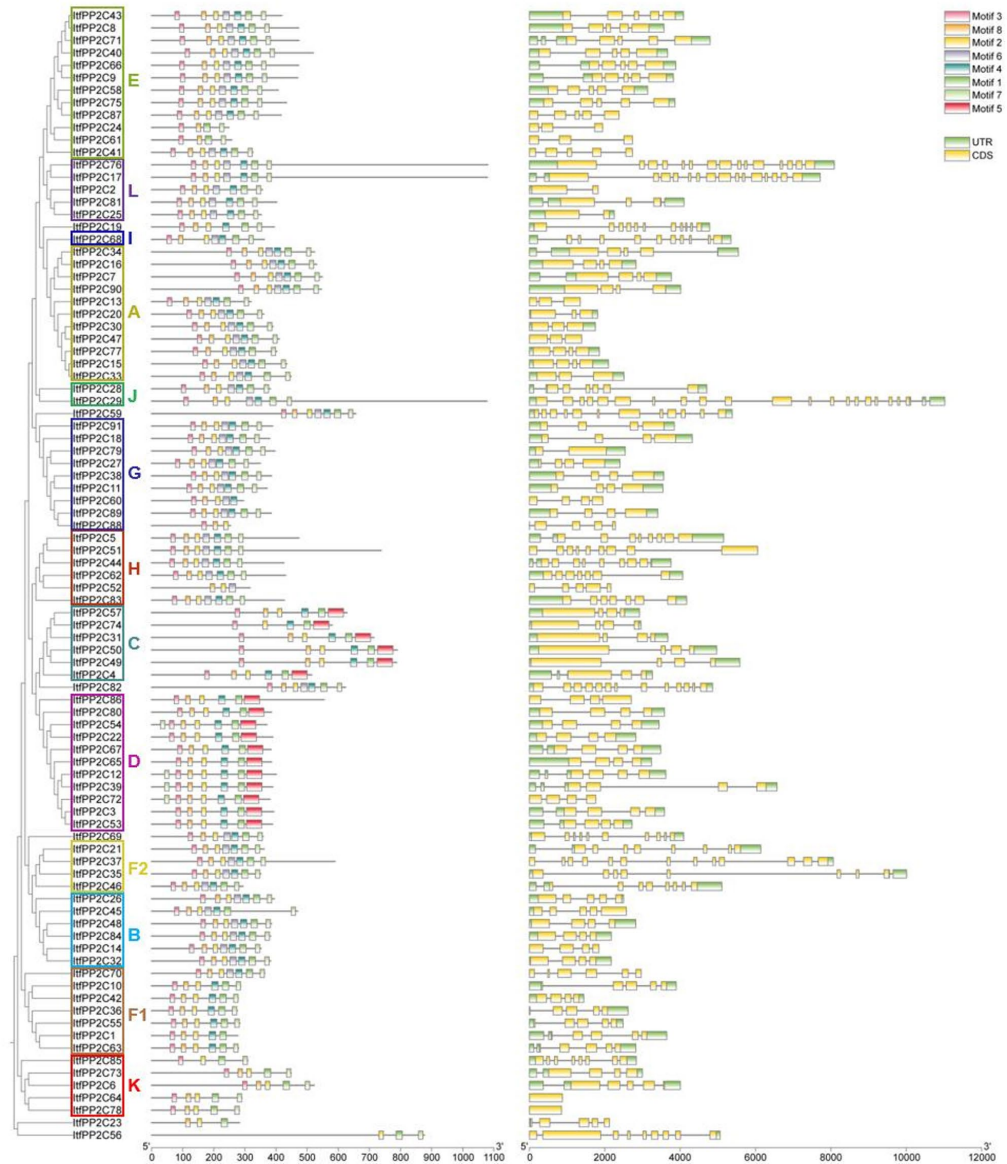
Conserved motifs and exon-intron structure analysis of *ItfPP2Cs.* Members belonging to different subfamilies were represented by different colored boxes. The eight conserved motifs were shown in different colors. In the gene structure figure, the green boxes, yellow boxes, and black lines represent UTRs, exons, and introns, respectively

The online MEME tool was used to identify 8 conserved motifs among different groups of *ItfPP2C *proteins (Table 1, Fig. S1). *ItfPP2C* genes belonging to the same subfamily were similar in terms of motif distribution, suggesting that motif distribution might be related to gene functions (Fig. 4). Motif composition varied substantially between *ItfPP2C* subfamilies. Notably, motif 5 was unique to members of subfamilies C and D, most of which also had motifs 1, 2, 3, 4, and 8, but not motif 6. The members of subfamilies F1 and K also lacked motif 6; the exception was *ItfPP2C70*. A large proportion of *ItfPP2C* genes contained motifs 1, 2, 3, 4, 6, 7, and 8. Motif 2 was the most conserved motif, followed by motifs 1, 3, and 8, which were equally conserved. In addition, *ItfPP2C52* (subfamily H), *ItfPP2C88* and *ItfPP2C60* (subfamily G), *ItfPP2C85* (subfamily K), and *ItfPP2C24* and *ItfPP2C61* (subfamily E) lacked different types of motifs that were present in other members of the same subfamily, possibly because of base deletions during tandem duplication events.

**Table 1:** Conserved motifs in the amino acid sequences of *ItfPP2C*

Motif	Width multilevel	Consensus sequence
1	21	DEFLILASDGLWDVLSNZEAV
2	15	LYVANVGDSRAVLCR
3	15	HFFGVFDGHGGSGAA
4	21	VNGGLAVSRAIGDFYLKKYVV
5	50	PRNGPARRLIKAALQEAAKKREMRYSDLKKIDRGVRRHFHDDITVIVIFL
6	21	DHKPNRPDERERIEAAGGRVI
7	15	GSKDBITVIVVDLKS
8	15	KEAJLKAFLKTDEEF

The gene structural diversity revealed in this study (Fig. 4) may reflect evolutionary differences among *ItfPP2C* genes. There was a relatively broad range in the number of exons in *ItfPP2C* genes. With the exception of *ItfPP2C64* and *ItfPP2C78*, the *ItfPP2C* genes contained both exons and introns. These results imply that the evolution and functions of *ItfPP2C* gene family members may be related to the diversity in their motif composition, exons, and introns.

### Putative *ItfPP2C* promoter cis-acting elements

**Fig. 5:**
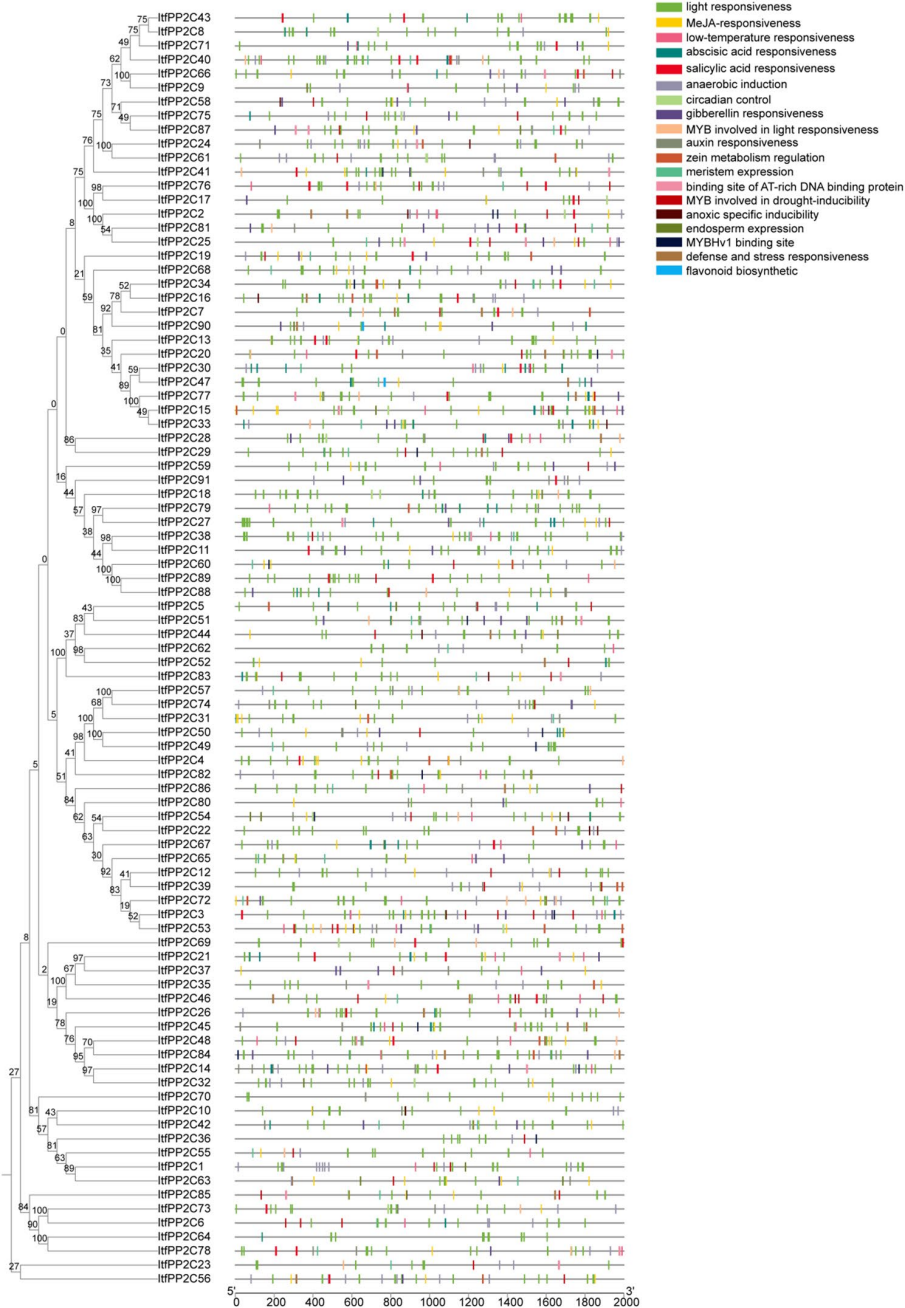
Cis-acting elements analysis in the promoters of *ItfPP2Cs*. A total of 19 cis-acting elements were identified in the promoters of the *ItfPP2Cs* genes

The promoter regions 2-kb upstream of the 91 *ItfPP2C* genes were screened for cis-acting elements using PlantCARE (Fig. 5). A total of 19 cis-acting elements were identified in the *ItfPP2C* promoters, with light-responsive elements detected in all promoters. Several cis-acting elements responsive to hormones, including ABA, methyl jasmonate, salicylic acid, gibberellin, and auxin, were also detected, indicating that these genes may be involved in plant hormone pathways. Additionally, many cis-acting elements associated with responses to various stresses, such as low temperatures, anaerobic conditions, drought (involving MYB transcription factors), and anoxic conditions, as well as plant defenses against biotic factors were identified in the examined promoters, suggesting the involvement of *PP2C* family members in biotic and abiotic stress responses. Moreover, a few regulatory elements were also detected, including those influencing circadian rhythms, zein metabolism, meristem expression, and flavonoid biosynthesis. These results indicate that *ItfPP2C* genes may be responsive to various environmental changes, thereby coordinating *I. trifida* growth and development.

### *ItfPP2C* expression in various tissues

**Fig. 6:**
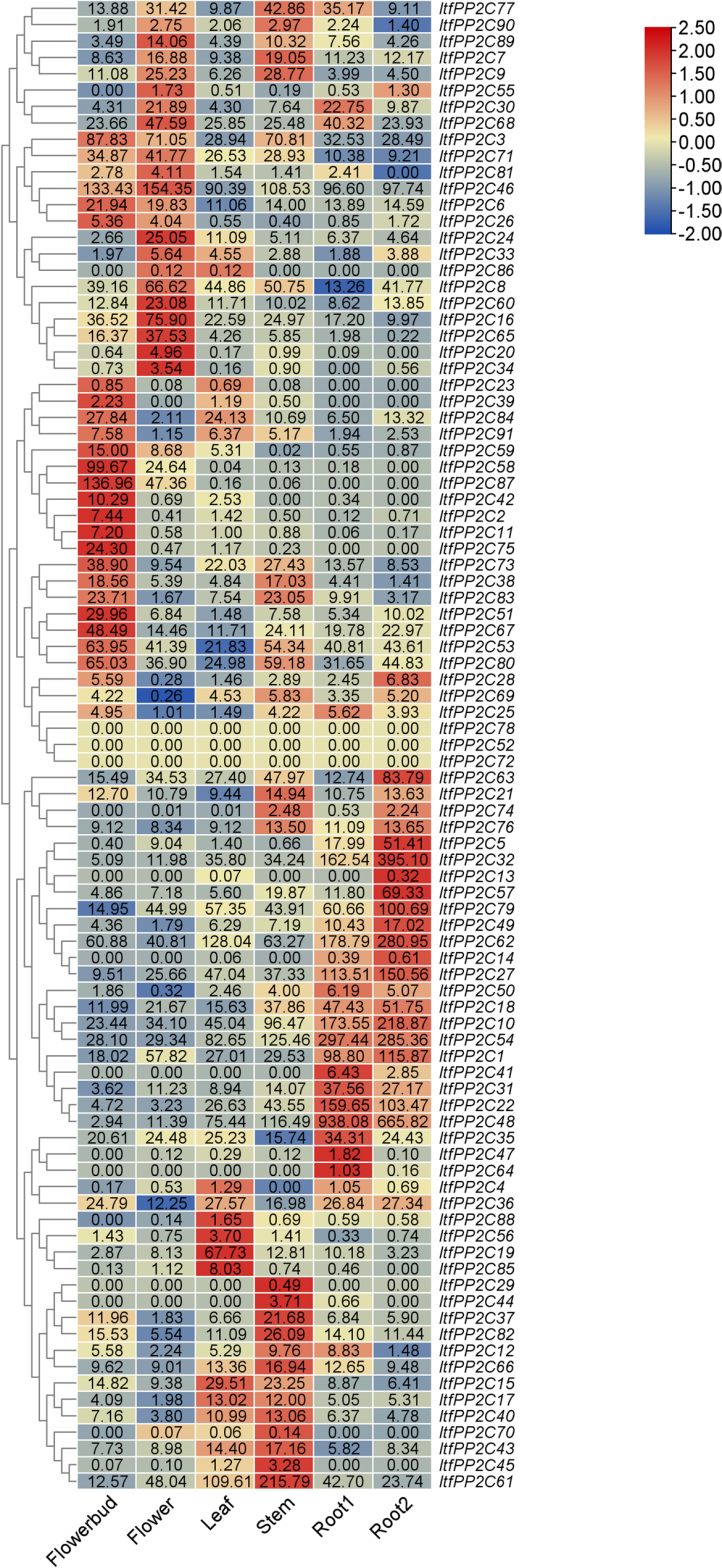
Gene expression analysis of *ItfPP2Cs* in different organisms. Gene expression patterns of *ItfPP2Cs* in the flowerbud, flower, leaf, stem, root 1, and root 2 of *I. trifida* as determined by RNA-seq. The log_2_ (FPKM) value is shown in the boxes

To analyze *ItfPP2C* expression patterns, RNA-seq data for six tissues (flower bud, flower, leaf, stem, root1, and root2) were analyzed, which revealed differences in *ItfPP2C* expression levels among tissues (Fig. 6). Nine *ItfPP2C* genes (*ItfPP2C68*, *3*, *46*, *53*, *80*, *62*, *10*, *54*, and *1*) were expressed at relatively high levels in all tissues. However, some *ItfPP2C* genes were expressed in specific tissues. For example, *ItfPP2C20* and *ItfPP2C34* were expressed in flower buds, *ItfPP2C42*, *2*, *11*, and *75* were expressed in flowers, and *ItfPP2C58* and *ItfPP2C87* were expressed in flower buds and flowers. Notably, these genes were expressed at almost undetectable levels in the other analyzed tissues. *ItfPP2C32*, *62*, *27*, *10*, *54*, *1*, *31*, *22*, *48*, *47*, *64*, and *41* were abundantly expressed in root1 and/or root2, but were expressed at low or undetectable levels in the other tissues, implying that they may be involved in root development. By contrast, *ItfPP2C29* was expressed specifically in the stem. Furthermore, *ItfPP2C47*, *64*, and *41* were not expressed in any of the examined tissues.

**Fig. 7:**
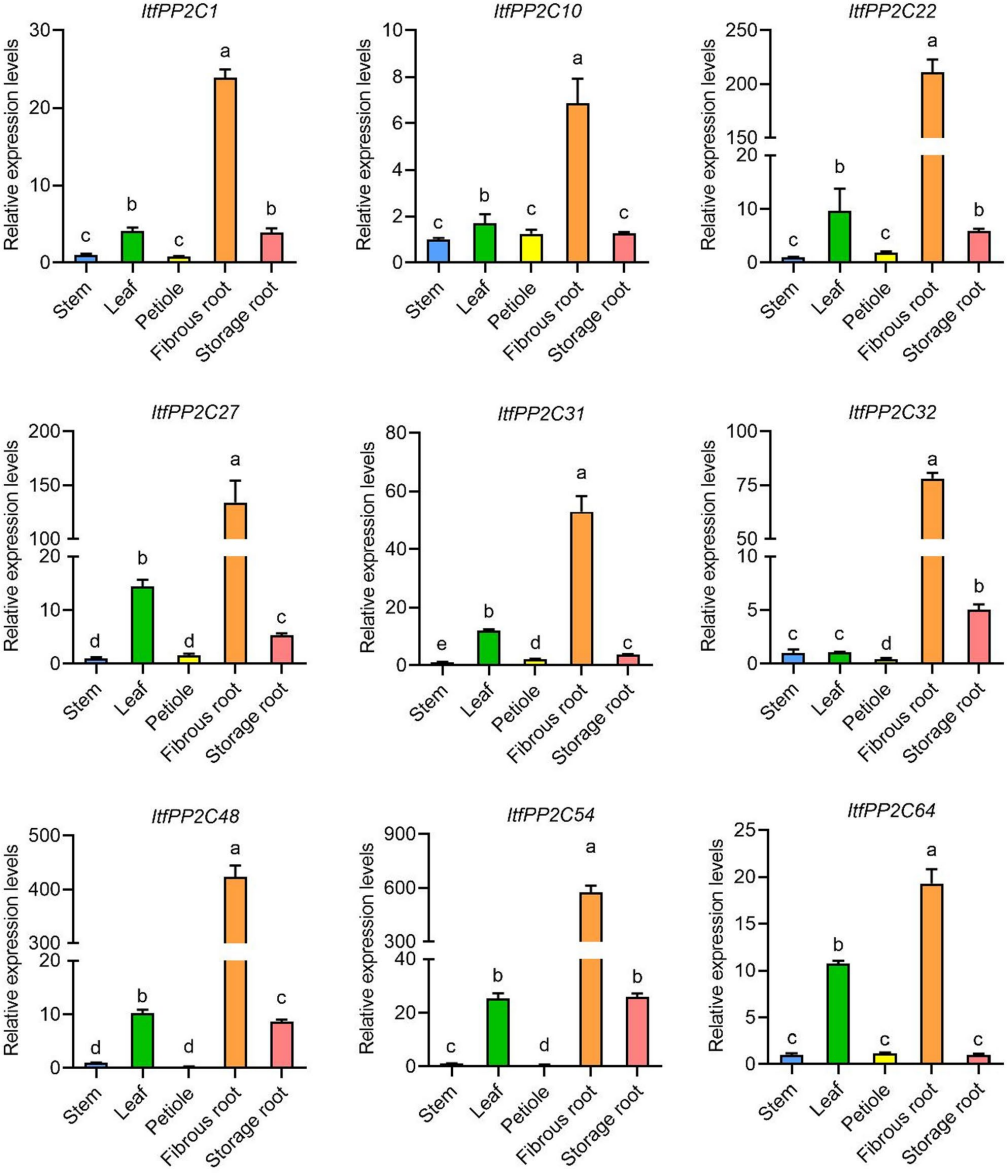
The expression patterns of root-preferential genes in different tissues were analyzed by qRT-PCR. Different letters indicate significant differences in relative expression levels among different tissues

To experimentally validate the tissue-specific expression patterns predicted by RNA-seq data, we selected nine genes that showed high expression levels in roots for qRT-PCR analysis across multiple tissues: fibrous roots, storage roots, stems, petioles, and leaves. As shown in Fig. 7, all nine genes exhibited the highest relative expression levels in fibrous roots. Notably, significant expression was also detected in storage roots, though generally at a lower level than in fibrous roots. Furthermore, a considerable level of expression was observed in leaves for several genes, suggesting their potential roles in this organ. Consistent with the RNA-seq data, their expression levels were very low or even undetectable in other tissues, such as stems and petioles.

### *ItfPP2C* expression levels in response to drought and a stem nematode infection

**Fig. 8:**
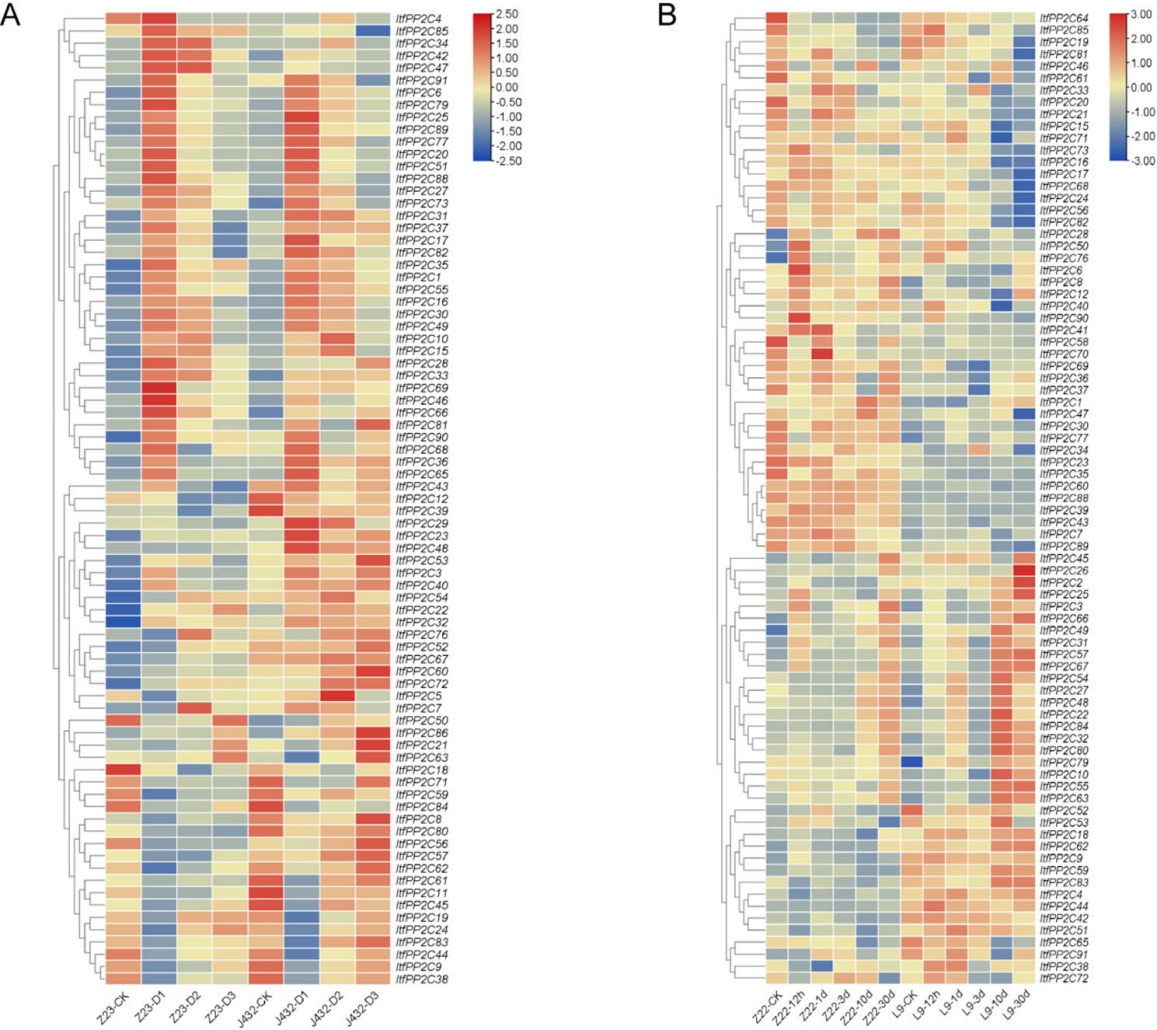
Expression patterns of *ItfPP2Cs* genes under drought and stem nematode stresses. **A** Heatmap illustrating the expression levels of *ItfPP2Cs* genes under drought stress in drought-sensitive variety, i.e., Jinong432 (J432), and drought-tolerant variety, i.e., Zhenghong23 (Z23) based on RNA-seq data. **B** Heatmap illustrating the expression levels of *ItfPP2Cs* genes under stem nematode stress in stem nematode-sensitive variety, i.e., Longshu9 (L9), and stem nematode-tolerant variety, i.e., Zhenghong22 (Z22) based on RNA-seq data

To explore the possible roles of *ItfPP2C* genes in biotic and abiotic stress responses, we analyzed RNA-seq data to determine their expression profiles under drought conditions and following a stem nematode infection. The expression levels of most *ItfPP2C* genes were upregulated after drought treatments (Fig. 8A). Previous research indicated that most *PP2C* genes in subfamily A encode proteins with negative regulatory effects on drought stress responses [42]. In the current study, the expression levels of most *ItfPP2C* genes in subfamily A, including *ItfPP2C7*, *15*, *16*, *20*, *30*, *33*, *34*, *77*, and *90*, initially increased and then decreased after an exposure to drought stress. Specifically, *ItfPP2C15*, *16*, *30*, and *77* expression levels increased significantly under drought conditions, implying that they may be important candidate genes related to drought resistance in *I. trifida*.

*ItfPP2C* expression levels were compared between Zhenghong22 (Z22; resistant to stem nematode infections) and Longshu9 (L9); susceptible to stem nematode infections) plants at 12 h and 1, 3, 10, and 30 days after stem nematode infections (Fig. 8B). In response to stem nematode-induced stress, the expression levels of most *ItfPP2C* genes were upregulated, although this upregulation occurred at the later time points for a few genes. *ItfPP2C90* expression was significantly induced in Z22 and L9 at 12 h, implying that it may be associated with plant defense responses to stem nematode infections. The expression of some *ItfPP2C* genes was downregulated or almost unchanged in Z22, but significantly upregulated in L9; these genes included *ItfPP2C30*, *77*, and *89*, which are strongly considered to be associated with sweet potato resistance to stem nematode infections. Interestingly, *ItfPP2C30* and *ItfPP2C77* were identified as candidate genes contributing to plant responses to drought stress and stem nematode infections. The diversity in expression patterns among *ItfPP2C* genes suggests that the encoded proteins may play specific roles in response to various stresses.

**Fig. 9:**
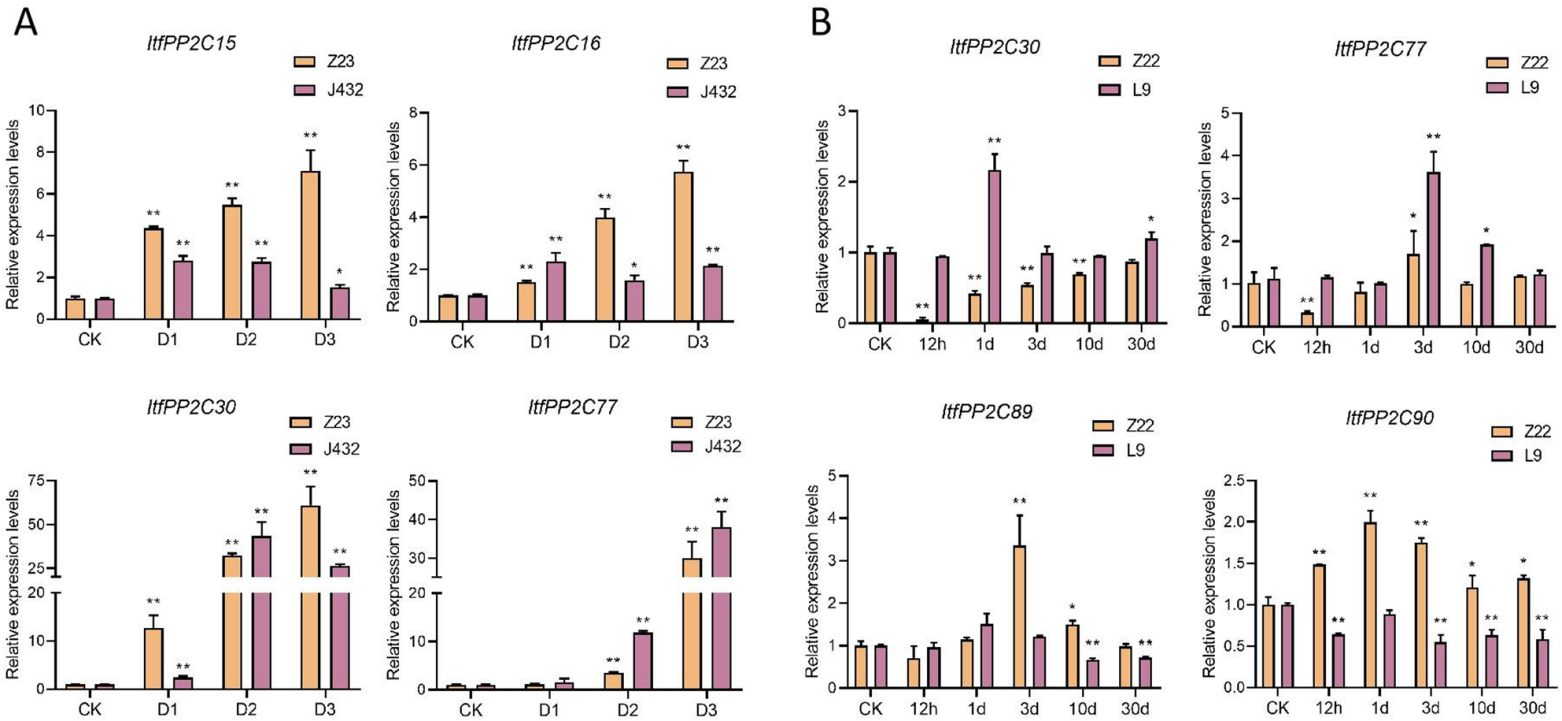
qRT-PCR analyses of key candidate genes under drought and stem nematode stress. **A** Relative expression levels of four candidate genes of drought-resistant cultivar Z23 and drought-sensitive cultivar J432 at different time points under drought stress. **B** Relative expression levels of four candidate genes of stem nematode-resistant cultivar Z22 and susceptible cultivar L9 at different time points post-inoculation. Data are presented as mean ± SD (*n* = 3). Asterisks indicate significant differences between the CK and different time points (*p < 0.05, **p < 0.01)

To validate the expression patterns of candidate genes under stress conditions, we selected all four identified key candidate genes for each treatment based on our RNA-seq data and performed qRT-PCR analysis under drought stress and stem nematode infection, respectively, with multiple time points examined (Fig. 9). Under drought stress, the expression of the four candidate genes in the drought-resistant cultivar Z23 gradually increased with the duration of treatment. In contrast, in the drought-sensitive cultivar J432, their expression levels generally exhibited an initial increase followed by a decrease, with the exception of *ItfPP2C77* (Fig. 9A). Notably, the expression of all candidate genes was significantly induced by drought stress, consistent with our RNA-seq data. For stem nematode infection, in the resistant cultivar Z22, the expression levels of *ItfPP2C30*, *ItfPP2C77*, and *ItfPP2C89* decreased at 12 h post-treatment. In the susceptible cultivar L9, the expression levels of these three genes generally exhibited a trend of first increasing and then decreasing, which was in line with the RNA-seq results. Compared with the control (CK), the expression of *ItfPP2C90* was upregulated in the resistant Z22 but downregulated in the susceptible L9 after infection (Fig. 9B). Collectively, these qRT-PCR results corroborate the RNA-seq findings and significantly enhance the credibility of these genes as key regulators in the response to drought and stem nematode stress.

### Proteins predicted to interact with ItfPP2C30 and ItfPP2C77

**Fig. 10:**
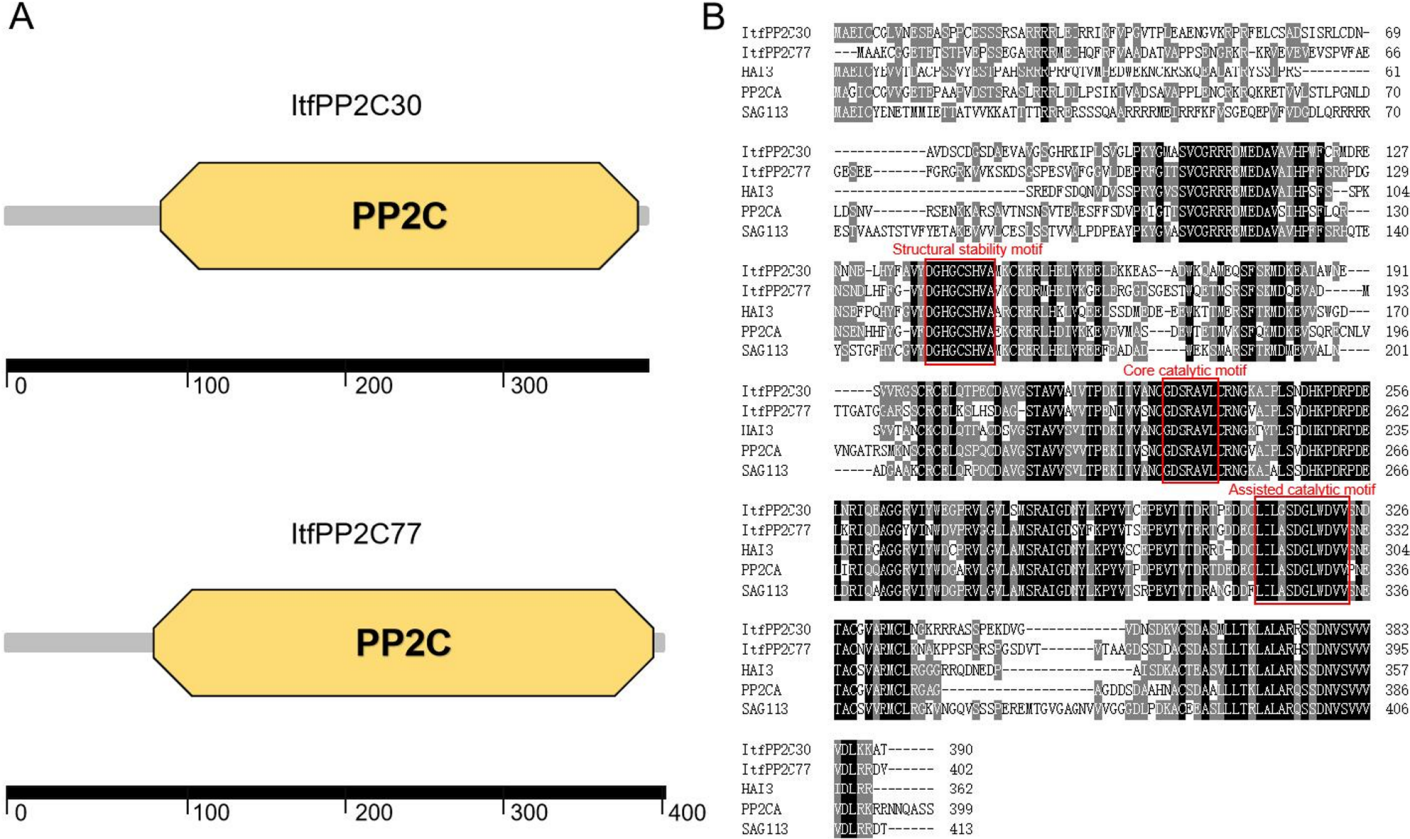
The conservative analysis of ItfPP2C30, ItfPP2C77 and their homologs in *Arabidopsis*. **A** ItfPP2C30 and ItfPP2C77 contain a conserved *PP2C *domain at amino acid positions 83–384 and 81–396, respectively. **B** Protein multiple sequence alignment of ItfPP2C30, ItfPP2C77, and HAI3, PP2CA, and SAG113 in *Arabidopsis*. The Red boxes represent conserved motifs

An analysis of protein sequences using SMART online software indicated that ItfPP2C30 and ItfPP2C77 contain *PP2C *conserved domains at amino acid positions 83–384 and 81–396, respectively (Fig. 10A). ItfPP2C30 and ItfPP2C77 protein sequences were examined using the Single/Multiple Proteins by Sequence module of SMART, which revealed that the top three proteins that matched ItfPP2C30 and ItfPP2C77 were the same (i.e., HAI3, PP2CA, and SAG113). An alignment of ItfPP2C30, ItfPP2C77, HAI3, PP2CA, and SAG113 sequences detected shared conserved motifs, such as GDSRAVL, indicating that these proteins may have similar functions (Fig. 10B).

**Fig. 11:**
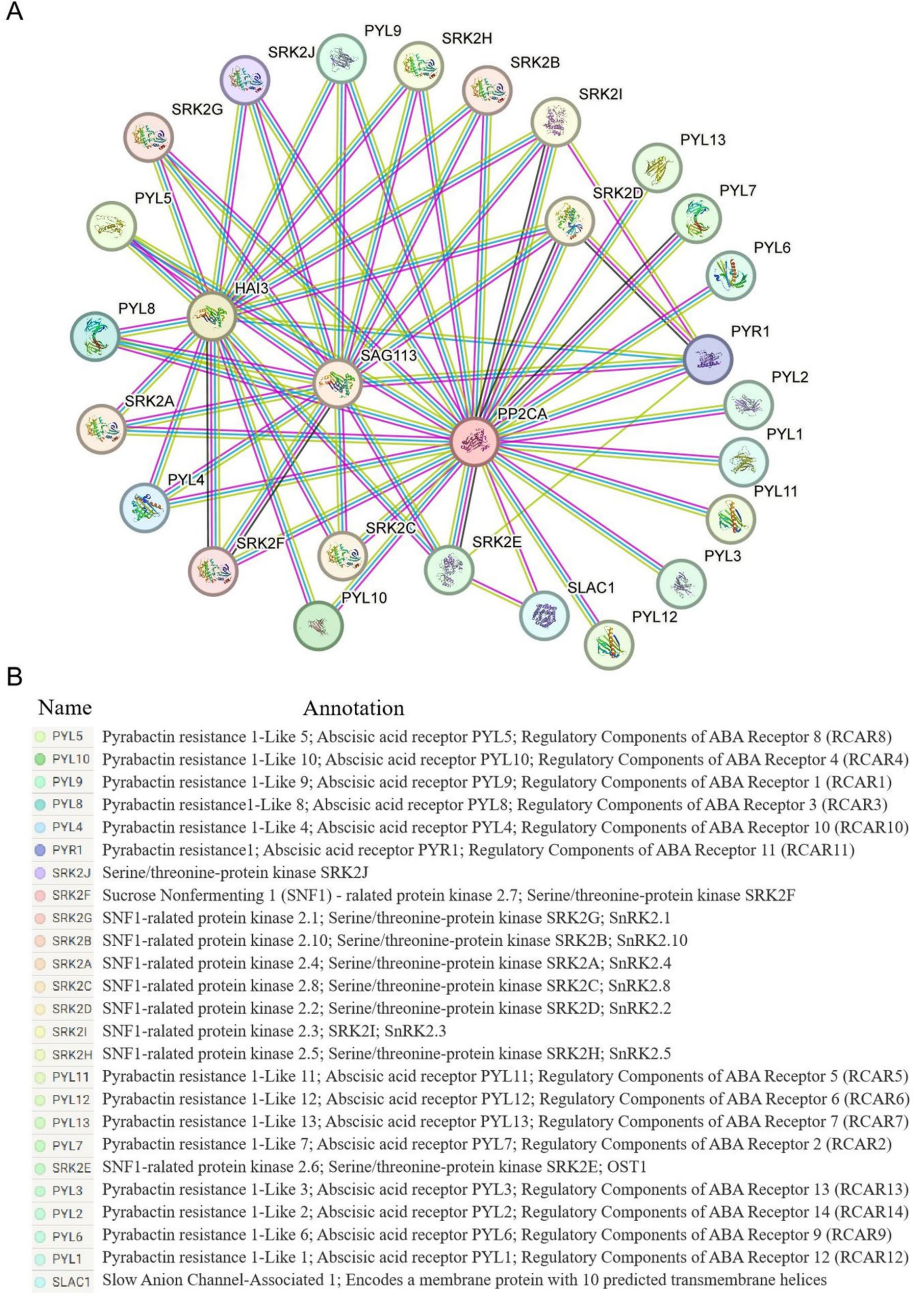
Prediction of interacting proteins of ItfPP2C30 and ItfPP2C77. **A** The predicted interactions network diagram of ItfPP2C30 and ItfPP2C77 from SMART website. The model organism *Arabidopsis thaliana* was used as reference. The top three proteins that match ItfPP2C30 and ItfPP2C77 are the same, namely HAI3, PP2CA, and SAG113. The blue and rose red lines represent known protein-protein interactions, where blue represents interactions from cured databases and rose red lines represent interactions from experimentally determined. The yellow line represents protein-protein interaction in textmining, whereas the black line represents co-expression of proteins. **B** The predicted interacting proteins and their functional annotations from the TAIR website

We selected HAI3, PP2CA, and SAG113 to screen for interacting proteins. As shown in Fig. 11, the line connecting two proteins represents a predicted interaction relationship, and most proteins are predicted to interact with HAI3, PP2CA, and SAG113 simultaneously, including ABA receptors (such as PYL4 and PYL5) and SnRK2 kinases (such as SRK2A/SnRK2.4 and SRK2B/SnRK2.10), which are likely important candidate interacting proteins (Table S3). Some PYL proteins, including PYL1, PYL2, PYL3, PYL6, PYL7, PYL11, PYL12, and PYL13, as well as the guard cell S-type anion channel SLAC1 only interacted with PP2CA (Fig. 11A and B, Table S3).

## Discussion

*PP2C *family members play essential roles in regulating plant growth, development, and stress responses [43, 44]. In recent years, the rapid development of bioinformatics tools has been accompanied by an increasing number of reports describing the whole-genome identification of *PP2C* genes in plants [1, 3, 45]. However, the characteristics of *PP2C *genes in *I. trifida* remain unclear. The genome-wide identification of *PP2C* genes in *I. trifida* has provided critical insights into the evolutionary and functional diversification of these phosphatase-encoding genes in a diploid relative of cultivated sweet potato. In this study, 91 *ItfPP2C* genes were identified, which is more than the number of reported *PP2C* genes in *A. thaliana* (80) and rice (78) [14], suggesting that the number of *PP2C* genes may be related to various factors, including genome size or whole-genome duplication events [46, 47]. The uneven chromosomal distribution of *ItfPP2C* genes may reflect gene duplication, which is a common mechanism for expanding stress response-related gene families in plants [48].

Notably, the retention of multiple *PP2C* gene subfamilies (A–L) in *I. trifida* suggests that *PP2C* genes were highly conserved in various species during evolution, highlighting their indispensable roles in plant development and evolution [14, 47]. Phylogenetic analysis revealed distinct functional specificity among the ItfPP2C subfamilies, showing significant homology with well-characterized *Arabidopsis* PP2C proteins. ItfPP2C77, identified as a key drought-stress candidate gene in our study, is most closely related evolutionarily to AtPP2C37 (AtPP2CA), a crucial negative regulator of the ABA signaling pathway. Among subfamily A members, ItfPP2C90 is highly homologous to AtPP2C77 (ABI2) and AtPP2C56 (ABI1), while ItfPP2C7 is highly homologous to AtPP2C16 (HAB1) and AtPP2C7 (HAB2). In *Arabidopsis*, ABI1 and ABI2 were the first *PP2Cs *discovered to function as negative regulators in ABA-related signaling pathways, participating in the regulation of stomatal closure, seed germination, and plant growth. Together with HAB1 and HAB2, they also act as negative regulators of drought and salt stress [49, 50]. In subfamily B, ItfPP2C14 and ItfPP2C32 are highly homologous to AtPP2C30 (PP2C5), a MAPK phosphatase that positively regulates seed germination and stomatal closure [28]. ItfPP2C27, a member of subfamily G, is highly homologous to AtPP2C49, a negative regulator of salt stress in *Arabidopsis* [51]. Among subfamily E members, ItfPP2C61 and ItfPP2C24 cluster in the same branch as AtPP2C73 (EGR2), which is involved in low-temperature stress responses [52]. In subfamily D, ItfPP2C53, ItfPP2C3, ItfPP2C39, ItfPP2C72, and ItfPP2C12 cluster in the same branch as FGT2, a protein associated with high-temperature stress responses [53]. These findings suggest that *PP2C *proteins in sweet potato that are highly homologous to those in *Arabidopsis* may have conserved functions, while functional divergence has occurred among different subfamilies.

Interestingly, *ItfPP2C19*/*23*/*56*/*59*/*69*/*82* were not clustered in any subfamily, similar to *AtPP2C8*/*31*/*44*/*54*/*57*/*70* in *A. thaliana* and *OsPP2C4*/*5*/*21*/*67* in rice. This may reflect the emergence of these genes during evolution, implying that some unclassified genes may have evolved specifically to satisfy developmental needs or confer stress resistance in plants [47]. The upregulated expression of *ItfPP2C30* and *ItfPP2C77* during stem nematode infections highlights their dual functions in biotic and abiotic stress responses. This parallels the dual roles of *A. thaliana* PP2C38 in modulating ABA signaling and responses to pathogen attacks [29], suggesting conserved mechanisms across species. An analysis of cis-acting elements revealed many stress-responsive motifs in *ItfPP2C* promoters, providing additional evidence of the involvement of *ItfPP2C* genes in hormone signaling and stress responses. Moreover, root-specific *ItfPP2C* expression implies that the encoded proteins modulate the root architecture under stress conditions, similar to *A. thaliana* PP2C.D6 and PP2C.D7, which affect root formation [54]. Conversely, the lack of *ItfPP2C47*, *64*, and *41* expression among tissues may reflect their functional redundancy.

The predicted interactions of ItfPP2C30 and ItfPP2C77 with PYL and SnRK2 proteins suggest that they may serve as hubs in ABA signaling pathways, similar to the *A. thaliana* PYL–*PP2C*–SnRK2 ternary complex, in which *PP2Cs *function as co-receptors for the deactivation of SnRK2s [25, 55]. The interaction with SLAC1, a key anion channel related to stomatal closure, indicates ItfPP2C proteins may have regulatory effects on osmotic stress responses [56]. However, the absence of direct interactions with immune receptors, such as FLS2 or CERK1, may reflect distinct pathways regulating biotic stress responses, possibly involving MAPK cascades [57].

While this study establishes a solid bioinformatics foundation for understanding the *PP2C *gene family in *I. trifida*, including their phylogenetic relationships, conserved motifs, and stress-responsive expression patterns, several limitations should be acknowledged. A primary constraint is the lack of direct experimental validation through transgenic approaches for key candidate genes such as *ItfPP2C30* and *ItfPP2C77*. In addition, although interactions within the PYL-*PP2C*-SnRK2 complex were predicted, experimental verification using methods such as yeast two-hybrid (Y2H) or bimolecular fluorescence complementation (BiFC) is still required. These approaches are essential for confirming the proposed functions of candidate genes in drought and nematode resistance, as well as their interactions with core components of the ABA signaling pathway. To address these limitations, a clear experimental plan has been developed for future research: (1) Generation of stable transgenic sweet potato lines overexpressing *ItfPP2C30* and *ItfPP2C77* to evaluate their roles in drought and pathogen stress tolerance; (2) Implementation of Y2H and BiFC assays to validate physical interactions between these ItfPP2Cs and key ABA signaling elements, including PYL receptors and SnRK2 kinases, thereby clarifying their mechanistic roles in stress response pathways.

## Conclusion

This study systematically identified and characterized 91 *PP2C* genes in *I. trifida* and then elucidated their phylogenetic relationships, conserved motifs, and stress response-related expression patterns. The considerable expansion of the *PP2C* gene family in *I. trifida* suggests its potential importance for stress tolerance. Key candidate genes for drought and nematode resistance, including *ItfPP2C30* and *ItfPP2C77*, are predicted to encode proteins that may interact with ABA signaling pathway components. According to their promoter cis-acting elements and expression profiles, these genes appear to contribute to adaptations to hormonal and environmental stresses. These findings provide insights into *PP2C* evolution in *Ipomoea* species, while also offering potential genetic targets for enhancing stress tolerance in sweet potato via molecular breeding. Future work should focus on the functional characterization of these candidate genes to better assess their utility for crop improvement strategies.

## Supplementary Information

Supplementary Material 1

Supplementary Material 2

## Data Availability

The sequence information of *Arabidopsis* PP2C family genes were collected from The Arabidopsis Information Resoure (https://www.arabidopsis.org/). The PP2C family expression data were downloaded from the Sweet Potato Genomics Resource (http://sweetpotato.plantbiology.msu.edu/generated). The datasets used and/or analyzed during the current study are available from the corresponding author on reasonable request.

## Abbreviations

PP2C: Protein phosphatase 2 C
PYL: Pyrabactin resistance 1-like
SnRK2: Sucrose non-fermenting 1-ralated protein kinase2
PPs: Protein phosphatases
ABA: Abscisic acid
MAPK: Mitogen-activated protein kinase
FPKM: Kilobase of exon per million fragments mapped
pI: Isoelectric point
UTR: Untranslated regions
CK: Control check
