# Supplementary figures and images for: Genome-wide identification and functional characterization of PP2C genes in the wild relative of sweet potato Ipomoea trifida
Source: BMC Plant Biol. 2025 Dec 29;25:1742. doi: 10.1186/s12870-025-07764-4 (PMC12752091; doi:10.1186/s12870-025-07764-4)

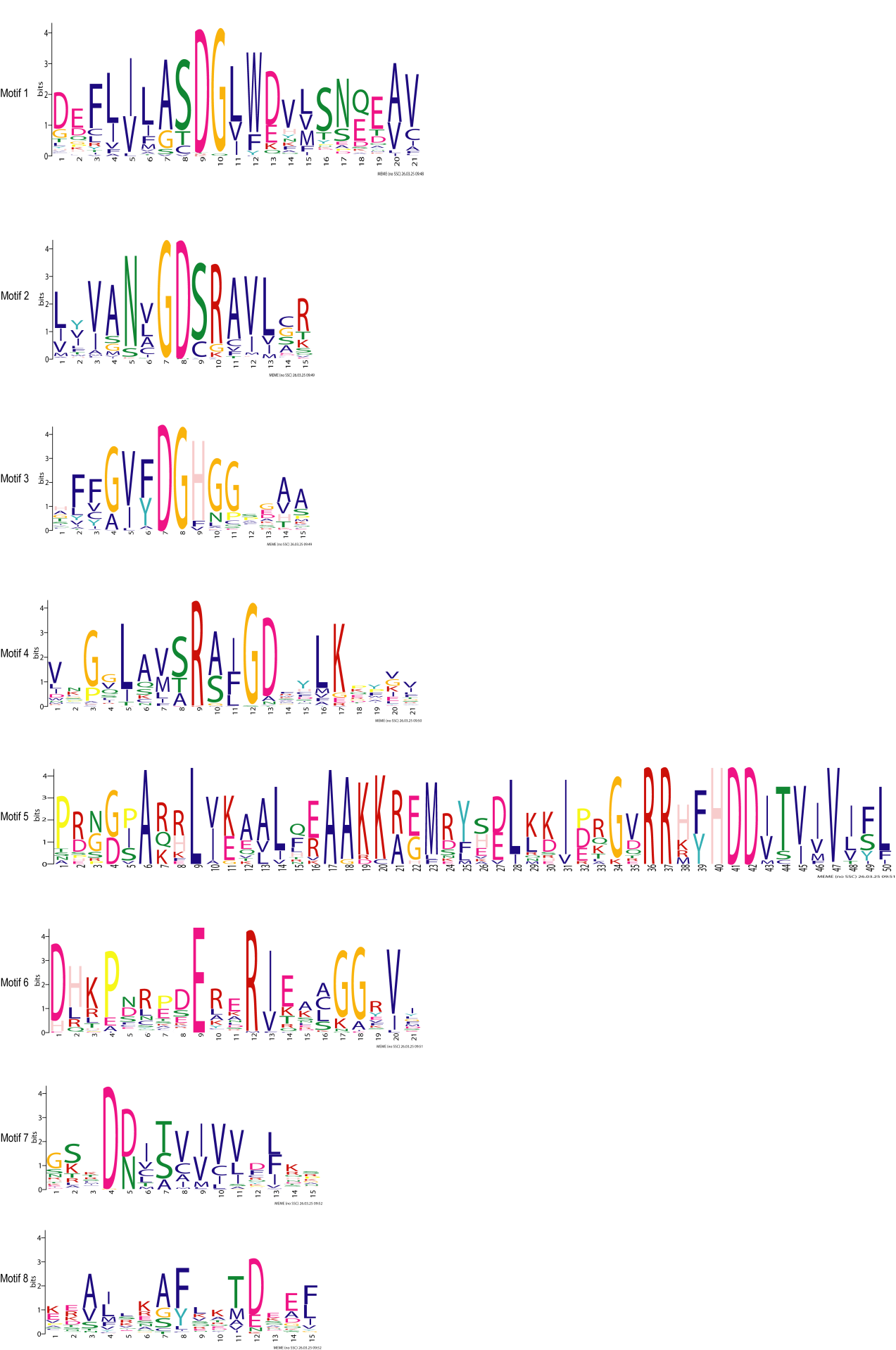


Fig. S1 Composition and distribution of conserved motifs in the protein sequences of the ItfPP2C.

Supplement: Supplementary file 2 — Supplementary Material 2 [file 12870_2025_7764_MOESM2_ESM.docx]
